# ChatGPT and large language models in orthopedics: from education and surgery to research

**DOI:** 10.1186/s40634-023-00700-1

**Published:** 2023-12-01

**Authors:** Srijan Chatterjee, Manojit Bhattacharya, Soumen Pal, Sang-Soo Lee, Chiranjib Chakraborty

**Affiliations:** 1 Institute for Skeletal Aging & Orthopaedic Surgery, Hallym University-Chuncheon Sacred Heart Hospital, Chuncheon-Si, 24252 Gangwon-Do Republic of Korea; 2 Department of Zoology, Fakir Mohan University, Vyasa Vihar, Balasore, 756020 Odisha India; 3 School of Mechanical Engineering, Vellore Institute of Technology, Vellore, Tamil Nadu India; 4 Department of Biotechnology, School of Life Science and Biotechnology, Adamas University, Kolkata, West Bengal 700126 India

**Keywords:** Large language models, ChatGPT, Orthopedics, Concern

## Abstract

ChatGPT has quickly popularized since its release in November 2022. Currently, large language models (LLMs) and ChatGPT have been applied in various domains of medical science, including in cardiology, nephrology, orthopedics, ophthalmology, gastroenterology, and radiology. Researchers are exploring the potential of LLMs and ChatGPT for clinicians and surgeons in every domain. This study discusses how ChatGPT can help orthopedic clinicians and surgeons perform various medical tasks. LLMs and ChatGPT can help the patient community by providing suggestions and diagnostic guidelines. In this study, the use of LLMs and ChatGPT to enhance and expand the field of orthopedics, including orthopedic education, surgery, and research, is explored. Present LLMs have several shortcomings, which are discussed herein. However, next-generation and future domain-specific LLMs are expected to be more potent and transform patients’ quality of life.

## Introduction

Artificial intelligence (AI) can be applied in various domains of medical science, such as imaging analysis and diagnosis; moreover, has been considered for enhancing the entire process of discovering and developing drugs, enhancing communication between doctors and patients, converting medical records (such as prescriptions) into text, and providing remote patient care [1–3]. Although AI technology has been available for many decades now, the last decade has forced countries to seek reasonable and acceptable rapid solutions in areas such as drug discovery, the delivery of medical services, and vaccine formulation development.

The recent COVID-19 pandemic has forced federal governments and multinational companies to explore quick and acceptable approaches, which may require integrating AI into the medical system. In November 2016, the USA Food and Drug Administration (FDA) approved the first AI-based diagnostic software. Approval was given to Arterys Inc. (San Francisco, CA, USA) for marketing a cardiovascular image analysis software for diagnosing cardiovascular diseases using a deep learning (DL) algorithm [4]. Subsequently, several AI-based medical products were approved [4, 5]. Zhu et al. analyzed the status of USAFDA-approved AI-enabled medical devices and found that the number of AI-based medical products has increased sharply over the past decade; additionally, they analyzed the trends and patterns of these devices [6, 7].

Physicians are adopting AI in the medical sciences, particularly in radiological diagnostics. Recently, Jha and Topol argued for the implementation of AI in the radiological diagnostics field in developed countries, such as the USA [8]. AI has made significant progress in medicine, particularly in cancer diagnosis, using IBM Watson's reliability, as demonstrated by Somashekhar et al. and Esteva et al., who used AI to identify skin cancer subtypes from clinical images [3, 9, 10]. In neurology, Bouton et al. developed an AI system for restoring movement control in quadriplegia patients [11], and Farina et al. tested an offline human/machine interface to control upper-limb prostheses using spinal motor neuron discharge timings [12].

In cardiology, Dilsizian and Siegel explored the potential of AI in diagnosing heart diseases using cardiac imaging [13]. Arterys has received FDA clearance for its Arterys Cardio DL application, which automatically generates editable ventricle segmentations from cardiac MRI images; this marks a significant step toward integrating AI into cardiology [4]. These examples highlight the diverse applications of AI in medicine, which are promising for enhancing patient care and medical outcomes. Therefore, the use of AI is experiencing a boom in medical sciences. Currently, AI-enabled ChatGPT is an addition to AI.

ChatGPT, an AI-enabled large language model (LLM), was developed using OpenAI (California, USA). It was released in November 2022 and became quickly popularized [14]. ChatGPT uses natural language processing (NLP) and DL to process vast amounts of data [15]. This chatbot has been applied in medical science, from essay writing and exam writing to diagnostic suggestions [16–18]. Researchers are exploring more medical applications of ChatGPT, and several articles have been published in different areas of medicine [19–23]. From students to researchers, everybody is exploring novel medical applications of ChatGPT to demonstrate its capability to perform a new functions.

Outpatient clinic letters are an important method of communication for most hospital specialists and general practitioners (GPs), and are significantly important in the medical field. These letters are the primary method of contact and communication between the hospital staff and GPs, and are often the sole record of consultation held by the outpatient department and hospital. Recently, ChatGPT was utilized to write clinical letters [24].

ChatGPT has also been applied to orthopedics. He et al. highlighted the significant medical applications of AI-enabled ChatGPT in orthopedics. They illustrated how ChatGPT/GPT-4 can help spinal surgeons by facilitating communication, streamlining data collection and analysis, assisting in surgical planning, and providing real-time support during endoscopic spinal surgery, thus ultimately improving patient care and outcomes when used appropriately and responsibly [25]. Thus, the applications of LLM and ChatGPT are being explored in various medical domains, including cardiology, nephrology, orthopedics, and ophthalmology, must be systematically documented.

In this light, this study mapped the different applications of LLMs and ChatGPT in orthopedics that have recently been explored. Additionally, various examples of LLMs and ChatGPT assisting orthopedic clinicians and surgeons in performing various medical tasks (such as orthopedic education, surgery, and research), and helping patients, clinicians, and researchers were explored to provide suggestions and guidelines. Finally, the drawbacks, limitations, and ethical concerns of ChatGPT were discussed.

## Potential of AI in orthopedics

The integration of AI in orthopedics offers the potential to improve patient outcomes and alleviate the workload of healthcare professionals. An exciting future prospect involves the creation of a digital twin, which virtually represents an individual and holds promise for advancing precision medicine by forecasting diseases, treatment results, and personalized preventive measures, even at the genetic level. This innovation has the potential to transform the fields of orthopedic surgery and medicine. AI applications in orthopedic surgery have the potential to identify problems with implants, such as misalignment and loosening; additionally, they can predict variables such as hospital stay, cost, functional recovery, and prognostic scores. However, the current state of AI technology necessitates a collective effort to transform theoretical concepts into practical clinical implementation. Thus, systematic and robust validation and reporting frameworks must be established to ensure the safe and effective adoption of AI in orthopedics [26]. The increasing significance of AI systems in the medical and orthopedic surgery fields is closely tied to the rapid growth of computer processing capabilities and cloud computing, and the continuous development and improvement of specialized software algorithms tailored for medical tasks. Given its substantial reliance on technologies such as medical imaging, which offer high sensitivity, specificity, and positive/negative prognostic indicators for managing orthopedic conditions, this field is exceptionally suitable for the utilization of machine-based integration for interpreting imaging studies and other applications [27].

## Large Language Models (LLMs) and ChatGPT

Several LLM applications are used by AI to generate various text-based content based on the provided textual instructions. Recurrent neural network (RNN) models were employed in earlier LLMs to comprehend text. RNN models are a type of artificial neural network that is typically employed in speech recognition and NLP. RNNs can comprehend the sequential characteristics of data, utilize patterns, and forecast. However, they face difficulties in processing lengthy text sequences. They struggle to retain information from the start of a paragraph and need help in simultaneously absorbing numerous words. With the development of transformer architectures, which are DL models, breakthroughs were made in 2017. These transformers enhance positional encoding and can simultaneously absorb numerous words while enabling parallel processing. LLMs constructed using these transformer topologies can include trillions of parameters [28–31].

An LLM is a sophisticated DL algorithm capable of handling diverse tasks in NLP. These models are trained on extensive datasets, which account for their considerable size. Consequently, they can identify, translate, forecast, and produce text and other forms of content. LLMs predict text based on the input by considering the context and varied meanings of words. They outperform traditional word-counting methods by offering a more authentic representation of the human language. LLMs are trained on vast amounts of text from online sources, which enables them to capture the nuanced expressions of mental states, generate human-like text, integrate information from diverse sources, engage in natural conversations, and simulate different linguistic styles and personas [32].

ChatGPT 3.5 is a sophisticated chatbot that generates conversational text using an LLM. Since its release to the public by OpenAI in November 2022, it has significantly influenced the field of higher education [33]. ChatGPT is built on the transformer model, a deep neural network that uses self-attention to understand the significance of different input data parts. It undergoes pretraining with extensive text data to learn the context and relationships in sequential data; this enables it to generate natural language responses. When users input text prompts, ChatGPT responds based on its understanding and the patterns from the training data. It is versatile, produces different response formats, from brief answers to detailed essays, and engages in conversations [2, 34].

LLMs such as ChatGPT have numerous applications in surgical science. One notable application involves utilizing them for writing tasks, which can significantly improve the effectiveness and efficiency of surgeons, scientists, and editors. Moreover, LLMs can be used to extract data and make clinical decisions, thus offering a promising avenue for their potential applications [35, 36]. This, in turn, demonstrates the trajectory of ChatGPT-type chatbots as a prospective model in orthopedic research, encompassing domains ranging from education to surgical practice.

## Evolution of chatbots

**Fig. 1:**
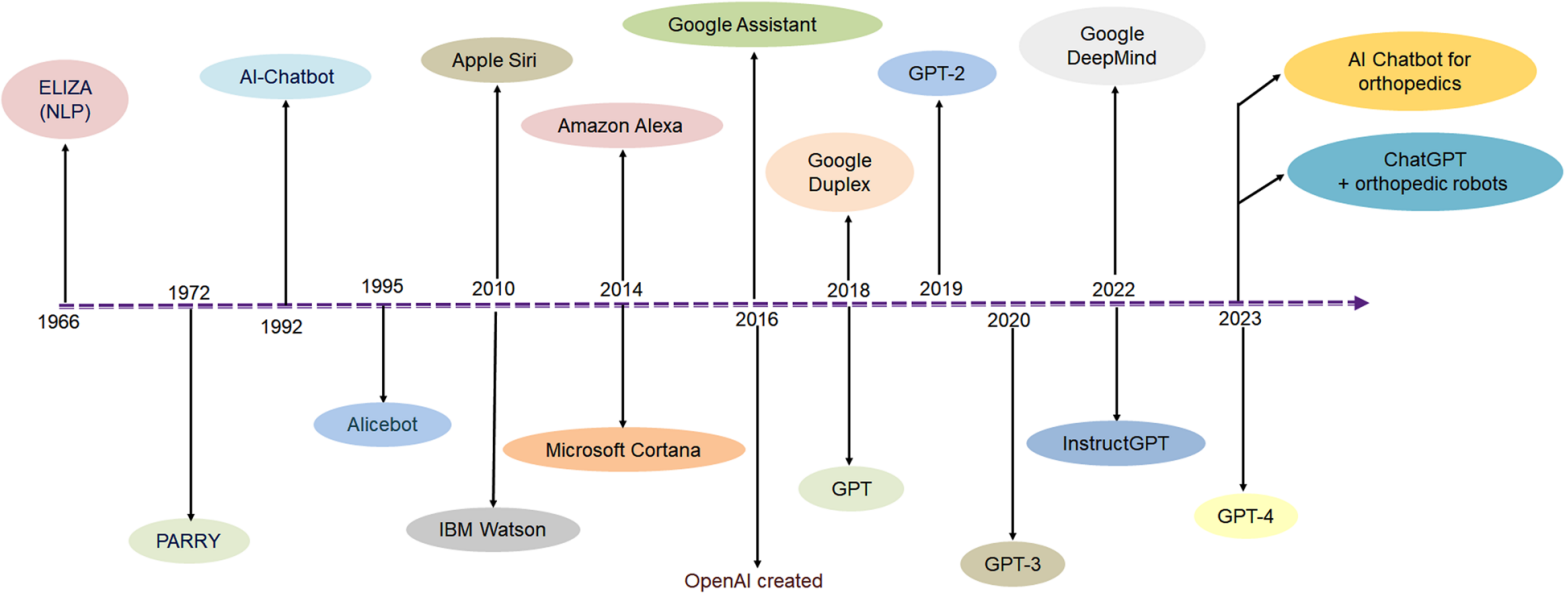
Timelines shows the different chatbots evolution

In 1950, Alan Turing first coined the term AI and explored its ideas. In this direction, his question was, “Can machines think?” [37]. The first chatbot, released under the name ELIZA, was based on NLP and was developed by Weizenbaum in 1966 at MIT [38]. It was considered the first program capable of attempting the Turing test, that is, to understand whether machines are able to think. Successively, its sensitivity was performed by a psychotherapist, who noted that it used simple pattern matching and a template-based response mechanism to match the conversational style [39]. Essentially, it operated on an applied pattern-matching interface [33]. Subsequently in 1972, an improved version of the chatbot ELIZA was released with added features; this version was called PARRY [38, 40]. Later in 1995, based on the pattern-matching algorithm and artificial intelligence markup language (AIML) for creating virtual assistance, a new chatbot, Alicebot, was released [19, 41]. Consecutively, several chatbots have been developed over time; these include the voice-driven digital assistants Apple Siri and IBM Watson in 2010, Amazon Alexa and Microsoft Cortona in 2014, and Google Assistant in 2016, which are all able to keep track of a conversation [29]. The newly developed chatbot is ChatGPT in its 3.5 version. Recently, OpenAI released a more improved form, GTP-4 (Fig. 1). Other organizations have launched competitors for ChatGPT and these include: Bard by Google, Bing Chat by Microsoft, Llama 2 by Meta, and Claude 2 by Claude.

## Applications of ChatGPT in orthopedics

**Fig. 2:**
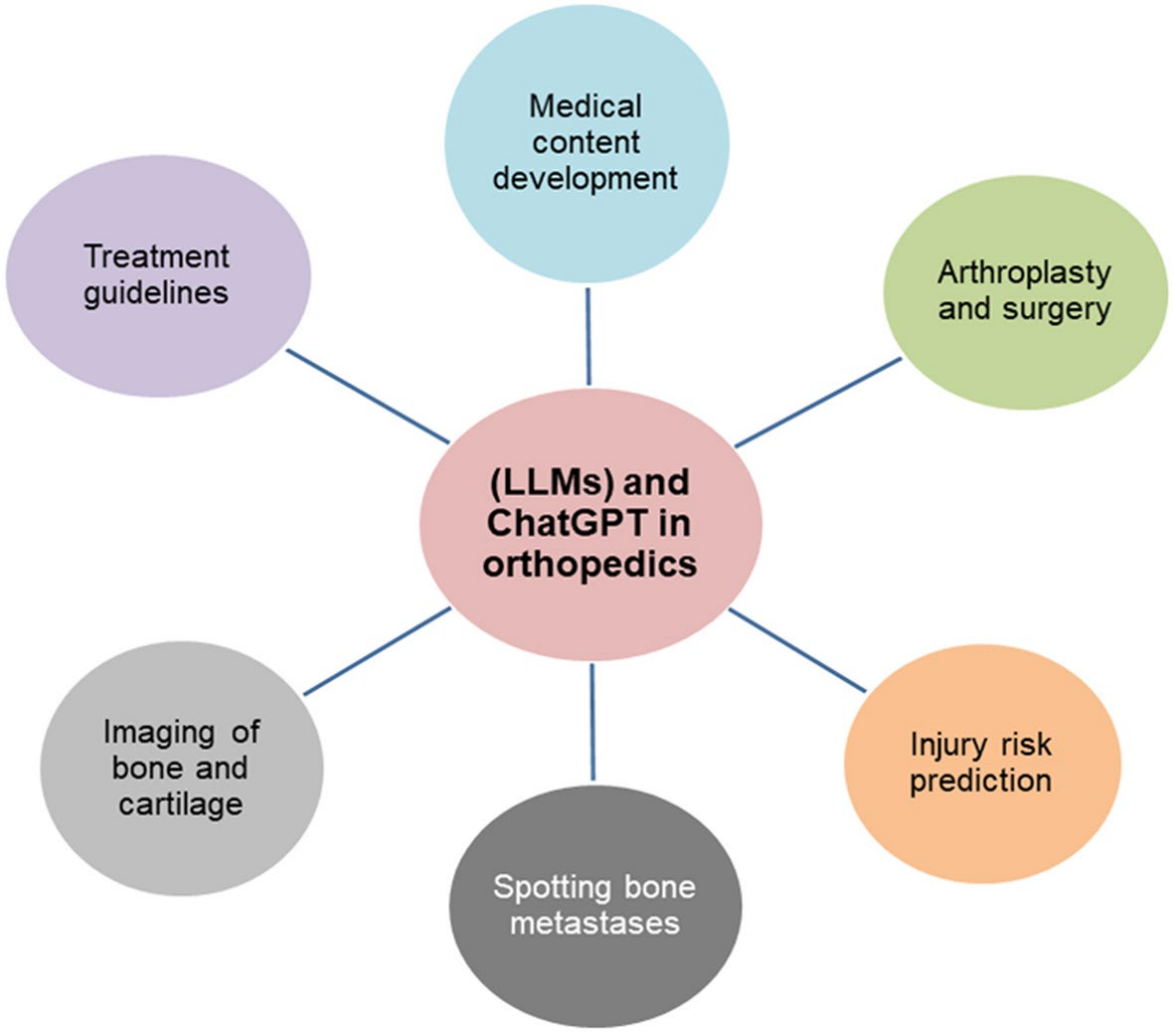
Schematic diagram shows ChatGPT’s different applications in orthopedic domain at medical science

Conversational AI is a revolutionary technology expected to advance further, thus offering the potential to enhance various aspects of our lives. Utilizing AI for tasks such as paperwork in the medical field can significantly improve efficiency [32]. Healthcare and medical industries can profit considerably from ChatGPT, a sophisticated language model. Essentially, ChatGPT can help doctors in various ways, including research, diagnosis, patient tracking, and providing medical instructions. However, ChatGPT has ethical drawbacks and restrictions such as biases, credibility, plagiarism, and copyright breaches. Thus, before using ChatGPT, these potential problems must be properly assessed and addressed. Future research should focus on determining how to reduce these drawbacks while keeping utilizing ChatGPT’s benefits in the healthcare and medical sectors [42]. AI technology has made significant advancements in the field of surgery and has already been used in some surgical operations. The adoption of ChatGPT has resulted in several surgical breakthroughs. Additionally, the accuracy of diagnoses can be improved by leveraging the ability of ChatGPT to analyze vast medical databases and assist in identifying rare conditions or recommending relevant tests. Furthermore, ChatGPT can be utilized to render surgical planning more efficient and safer by creating personalized preoperative plans. Postoperative care and rehabilitation can be strengthened by providing tailored recovery guidance and monitoring for patients through continuous communication and other improvements [11]. Recently, AI has been increasingly used to address problems in orthopedics, with various doctors and researchers utilizing this technology [43]. AI-enabled ChatGPT has been adopted to perform several functions in orthopedics, such as helping in education, suggesting medical care, and individual case analysis in surgery (Table 1). ChatGPT has several applications, such as orthopedic education, surgery, and research (Fig. 2), and these are discussed as follows.

**Table 1:** Application of Large Language Models (LLMs) and ChatGPT in orthopedics

Sl. No	Al tool used	Features	Category	Reference
1	ChatGPT	Helps in multiple aspects of spinal surgery, including precisely supporting surgeons, and with the aftercare of patients undergoing endoscopic spinal surgery due to lumbar disc herniation	Surgery	[44]
2	ChatGPT	Generates DL models for medical images to correctly detect bone metastases within the bone scans of numerous patients with cancer	Diagnosis	[45]
3	Deep neural network (AI) model	Used to diagnose bone metastasis; its evaluation is similar to that of human physicians	Diagnosis	[46]
4	AI-chatbot (ChatGPT)	Used to track activities, evaluate diagnostic images, predict the risk of injury, etc.	Diagnosis	[47]
5	ChatGPT	Applied to assess the section 1 of the fellowship of the Royal College of Surgeons (Trauma & Orthopedics) examination. It justified the role of ChatGPT in medical service provision and education	Education	[48]
6	ChatGPT	Assists orthopedic practitioners by providing rapid access to large amounts of medical information, research papers, and accurate treatment guidelines. Additionally, it supports clinicians in making correct diagnoses, choosing suitable treatment options, and developing personalized care plans for patients	Research	[49]
7	ChatGPT	Provides support for queries related to total knee arthroplasty and total hip arthroplasty by question type and topic to provide credible information	Surgery	[50]
8	LLMs and ChatGPT	Used to investigate the orthopedic surgery board examination (American Board) by challenging performance and knowledge that are equivalent to that of a first-year orthopedic surgery resident	Education	[51]

### Implication of ChatGPT in orthopedic education

A range of viewpoints has been expressed in response to ChatGPT in the academic and scientific community, essentially reflecting the ongoing discussion on the benefits and limitations of cutting-edge AI technologies [52–55].

#### Writing assignment

ChatGPT and other LLMs are useful for conversational and writing assignments as they increase output accuracy and productivity [56]. Hernigou and Scarlat suggested that ChatGPT could be a valuable asset in orthopedics. They explored various roles for ChatGPT, such as acting as author, reviewer, publisher, and editor, and its potential to address numerous questions related to the technology [49]. LLMs are used in ChatGPT, a general-domain AI model that has attracted considerable interest.

#### Performance in orthopedic examinations

Lum investigated how well the ChatGPT LLM responds to questions on orthopedic in-training examinations [36]. Reportedly, of the 400 OITE (Orthopaedic In-Training Examination) questions selected, 48% (193 questions) were excluded because they contained non-text data, such as images or figures, which could not be processed by the text-based LLM chatbot. To avoid memory retention bias, each of the 212 text-based questions was administered during separate chat sessions. The authors compared the performance of ChatGPT to that of various degrees of orthopedic surgery residents and found that ChatGPT could accurately respond to 47% of the questions; however, as the complexity of the questions increased, so did its performance. According to their results, ChatGPT can succeed in written boar for orthopedic surgery. Essentially, its performance and knowledge were on par with those of an orthopedic surgery resident in their first year. Thus, they suggest that AI such as ChatGPT may serve as an additional tool for orthopedic learning and education, particularly in knowledge- and interpretation-based inquiries [57]. In addition, Cuthbert and Simpson noted that ChatGPT cannot use sophisticated judgment and complex reasoning required to perform critical evaluations. ChatGPT had a score of 35.8%, which is 30% lower than that of the Trauma & Orthopedic Examination (FRCS) pass rate. Moreover, this score is 8.2% lower than the optimal score secured by a human applicant across all training levels [28].

#### Instructional model for orthopedic residents

The use of ChatGPT in the instruction of orthopedic residents during their rotation in orthopedic trauma has been noteworthy because it has produced a more engaging, intelligent, and approachable learning environment. Consequently, standards of patient care and education have improved. Thus, to enhance healthcare professionals’ learning opportunities, ChatGPT should be used in other fields, such as medicine and healthcare education [58].

### Implications of ChatGPT in orthopedic surgery

#### Guidelines in orthopedic surgery

ChatGPT and other language models have several potential applications in orthopedic surgery. These applications cover a plethora of functions, such as producing and improving textual content, extracting useful information from data, and supporting clinical decision-making. These models can help surgeons in several areas of their professions, including composing assignments, locating pertinent information, and handling patient-related issues [35]. An LLM can create notes far more quickly compared with manual note-making, while closely adhering to guidelines; these notes are well received by both surgeons and patients. These operative notes are even better with the addition of hyperrealistic graphics created by AI, which is possible in ChatGPT Plus with the DALL.E 3 feature. Although AI cannot entirely replace human input, these findings demonstrate the potential of AI in the medical field and present opportunities for future development [59]. Some applications of ChatGPT in orthopedic surgery are discussed below.

#### Disease diagnosis in assisting surgeons

**Fig. 3:**
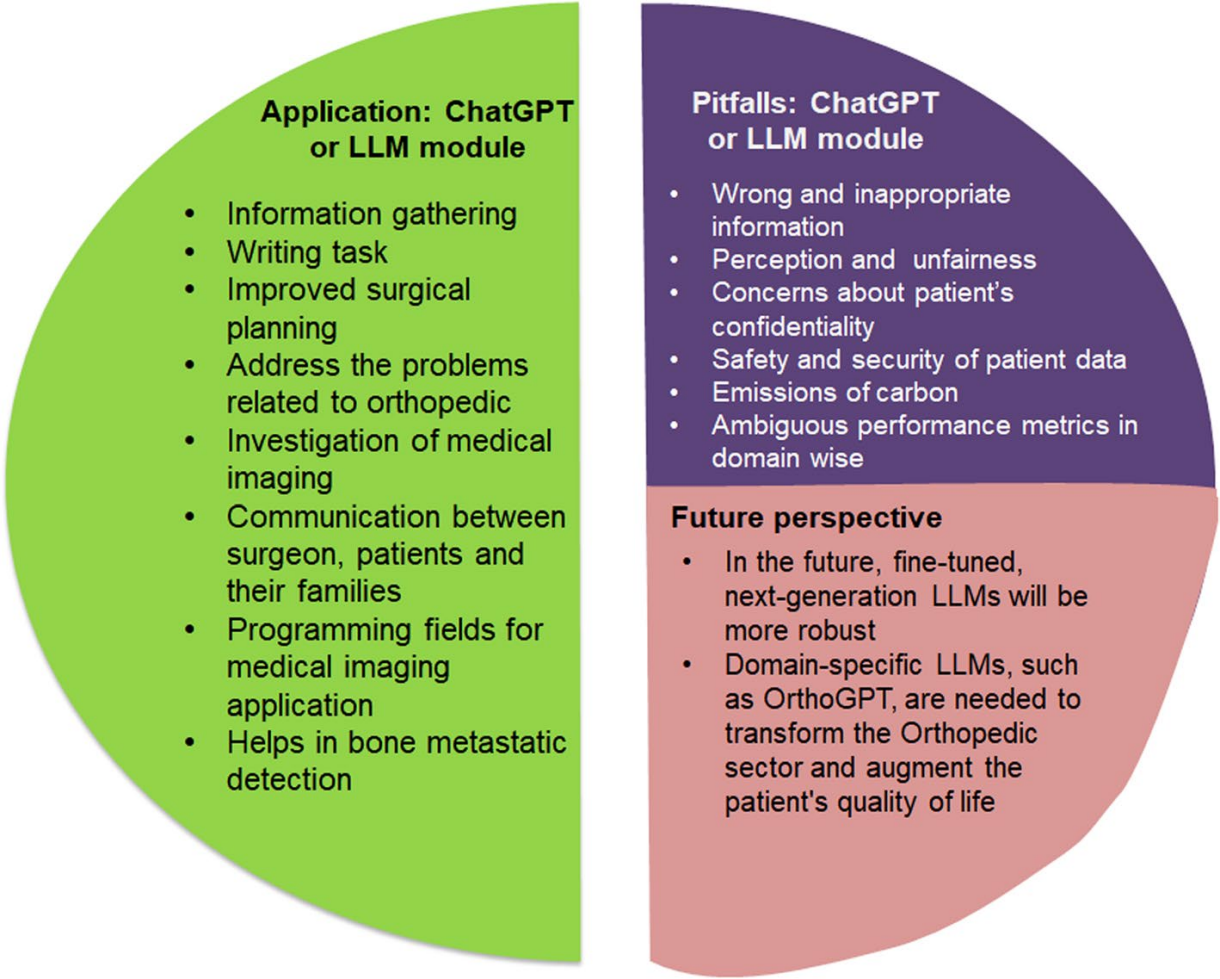
LLMs and ChatGPT in orthopedics. Applications in medicine, especially orthopedics, and its concern and future perspective in clinical and surgical orthopedics

The detection and prediction of osteoarthritis and imaging of bone and cartilage are among the areas of spine disease that have been the subjected of significant research, according to Cabitza et al. [42]. Medical imaging data has become the primary source of input data in numerous studies [43]. He et al. examined the potential advantages of utilizing ChatGPT in various aspects of spinal surgery, particularly in assisting surgeons in the aftercare of patients undergoing endoscopic spinal surgery for lumbar disc herniation. This AI-powered chatbot can enhance communication between surgeons, patients, and their families; simplify the gathering and evaluation of patient data; and assist in the preoperative preparation phase (Fig. 3). By providing immediate surgical navigation details, monitoring of physiological markers, and guidance for postoperative recovery, ChatGPT has the potential to enhance intraoperative assistance.

This study explored the multifaceted utility of ChatGPT/GPT-4 for spinal surgeons, including in preoperative patient communication, surgical planning, intraoperative assistance, rehabilitation guidance, and data management. Additionally, the potential benefits of using ChatGPT in these areas were illustrated, with emphasis on its role in enhancing patient care and surgical outcomes. However, this underscores the importance of maintaining data security and responsible use [25]. Without the need for sophisticated programming knowledge, doctors with basic programming skills can also use ChatGPT to create tailored DL models for medical imaging applications, such as spotting bone metastases in bone scintigraphy.

Son et al. used ChatGPT to build a DL model, thus demonstrating the possibility of leveraging AI chatbots such as ChatGPT to develop DL models for medical images [60]. These findings were promising, as their model demonstrated the potential for correctly detecting bone metastases in the bone scans of 4,626 cancer patients. Statistical analysis revealed that the model area under the curve (AUC) was 0.8156, which focused on enhancing data exclusively for positive cases [31]. Additionally, although slightly less impressive than the results of earlier trials, the diagnostic performance of the model, with 56.0% sensitivity and 88.7% specificity, was notable [60].

Examining certain instances in more detail revealed the dominance of AI over traditional medical intervention. For example, Zhao et al. utilized a deep neural network to create an AI model using a dataset of 12,222 instances of 99mTc-MDP bone scintigraphy [7]. The ability of the model to diagnose bone metastasis was evaluated and found to be similar to that of individual human physicians. Moreover, when an AI model was used to assist interpretation, it enhanced the diagnostic sensitivity and accuracy of human physicians. The diagnostic efficiency was represented by the receiver operating characteristic (ROC) curve, which showed values of 0.988 for breast cancer, 0.955 for prostate cancer, 0.957 for lung cancer, and 0.971 for the different cancer categories. This AI model also demonstrated its usefulness to nuclear medicine practitioners by delivering prompt and precise assessments of cancerous bone metastasis, which ultimately proved advantageous [50, 61].

#### Weight-bearing computed tomography (WBCT)

Weight-bearing computed tomography (WBCT) is a promising method for evaluating different lower-extremity deformities and ailments, such as knee osteoarthritis, ankle arthritis, progressive collapsing foot deformity, and hallux valgus. WBCT provides more accurate bone-positioning measurements than conventional weight-bearing radiographs and non-weight-bearing CT scans. Recent advancements in this area focus on a three-dimensional approach, utilizing automated joint mapping and machine learning (ML)-based segmentation techniques. AI-enhanced ChatGPT can be used in these applications [47].

### Implications of ChatGPT in orthopedic research

#### Assistance in orthopedic research

LLMs can assist researchers in generating clear and concise text, distilling large volumes of data, and performing various language-related tasks [32]. ChatGPT has made its way into scholarly writing, appearing in both preprints and published works. Although this undoubtedly benefits authors from various backgrounds, its advantages and disadvantages must be analyzed in the context of medical research [62]. The widespread use of ChatGPT has significantly disrupted the scientific community and sparked debates about the moral ramifications of using artificial intelligence (AI) to write scientific papers that could impact the decisions of policymakers, scientists, and medical professionals [63, 64]. However, whether to allow “it” to be a co-author on scientific publications has provoked heated debates [53, 65].

#### Domain-specific ChatGPT in orthopedic research

The exploration of several domain-specific ChatGPT indicates that they have the potential to significantly enhance orthopedic research, assisting in tasks ranging from literature review and data analysis to hypothesis generation and conclusions. However, the specific potential challenges and limitations associated with the use of ChatGPT in orthopedic research must be acknowledged. The model’s responses may require more context-specific knowledge and expertise from experienced orthopedic specialists to avoid errors or incomplete information.

## Drawbacks and limitations

Whether AI should be implemented into decision support systems has been a topic of discussion for several years [66]. As AI is increasingly used in radiology and orthopedic traumatology, several areas will be of great value in the future [4]. As is generally acknowledged, using AI in radiology and other image-based fields increases diagnostic precision [56]. However, before being included in clinical decision support systems, these tools must be carefully analyzed and interpreted [67, 68]. In addition, ChatGPT has identified many barriers and limitations affecting the successful incorporation of AI into surgical practice. These include lack of data availability, ethical concerns, complexity of surgical procedures, opposition to change, and budgetary limitations. These elements align with previous studies and scholarly discussions in this area, thus providing a thorough picture of the potential barriers to the practical application of AI in surgery [34, 40]. Moreover, concerns regarding the potential bias in ChatGPT resulting from training datasets need to be addressed. These biases could limit their skills and result in factual mistakes, a worrying condition known as hallucinations, in which results seem scientifically logical but may not be accurate. In addition, security concerns and the possibility of cyberattacks involving the spread of false materials via LLMs must be considered [60].

ChatGPT has attracted attention for several issues that could arise from the broad use of AI in surgical settings. These concerns include job losses, moral reasons, legal concerns, dependence on technology, and financial ramifications. These issues highlight the importance of carefully considering AI’s potential effects and consequences in surgery, and developing solutions for any problems that may arise [30]. However, the LLMs and ChatGPT provided inappropriate content and information (Fig. 3). Therefore, an AI-based LLM model should be adequately trained with the appropriate medical content to provide error-free and accurate medical information.

## Ethical concern

Following the pandemic, medical chatbots have made considerable strides as patient communication tools. Medical ethicists have accelerated the development and application of ethical standards to control transformative innovation. Medical chatbots face several legal and ethical difficulties that need to be addressed and resolved [48]. Owing to ChatGPT’s lack of widespread adoption in other medical applications, most scholarly research is focused primarily on ethical issues surrounding its use in medical contexts. Although ChatGPT reduces plagiarism, it requires human editing. Additionally, the validity of works produced by ChatGPT for personal remarks or letters of reference is questionable [5]. Additionally, AI-generated text must be ensured not to violate any existing copyrights before it is used for commercial purposes. Publishers and preprint servers claim that ChatGPT cannot be regarded as a study author because it is unable to assume responsibility for the veracity and legitimacy of scientific research, according to the Nature news team [13]. The ethical implications of ChatGPT for users must be investigated continuously and methodically. Thus, international collaboration must be promoted to establish a global ethical framework controlling the use of ChatGPT as a medical chatbot. The effective resolution of cybersecurity, privacy, seamless integration, and data content challenges relies heavily on the cooperation of diverse stakeholders, including medical practitioners, patients, hospital delegates, and computer security experts. Their joint efforts should concentrate on developing regulations for patient data ownership and security [69]. Thus, by addressing its difficulties and moral consequences, researchers can ethically use AI to further human understanding and knowledge. This strategy enhances the performance, usability, and overall user experience of ChatGPT and related conversational AI models across a range of domains and applications [70].

## Conclusions

The cutting-edge language models have expanded the possibilities for medical professionals and patients, altered surgical procedures, and increased education. It has developed into a priceless resource that provides immediate access to the information and experiences of orthopedic students and professionals. It can read intricate medical literature, examine patient data, and provide suggestions based on solid facts. Although there is great potential for using AI models such as ChatGPT in orthopedic research and surgery planning, several important issues must first be addressed. These include ensuring data privacy, mitigating bias, and establishing responsibility. By doing this, moral norms can be respected and egalitarian healthcare practices can be encouraged while utilizing the full potential of these AI technologies in orthopedics. For AI models, such as ChatGPT, continuous updates and enhancements are required to continue to be effective in orthopedic research. AI models must be continually trained on the most recent information and developments as the field of orthopedics develops with new techniques, treatments, and research. These models need to be kept current to provide orthopedic surgeons with precise and up-to-date advice. The focus must be on diminishing the growing gap between AI, LLM technological capabilities, and orthopedic professionals, such as doctors, surgeons, and researchers. Simultaneously, orthopedic doctors and surgeons must adopt next-generation LLMs to obtain a better clinical perspective. Thus, the primary objective of this study was to examine the diverse applications of ChatGPT and LLMs in the field of orthopedics, including education, surgery, and research. Further, this study sought to illustrate how these advanced language models can be utilized to enhance orthopedic practices, from facilitating education and surgical planning to supporting scientific research and data management. This article was written for healthcare service providers, doctors, patients, researchers, and students involved in orthopedic research. Although LLMs and ChatGPT may not be able to replace doctors, they can provide satisfactory information, knowledge, and guidance, which are of great assistance to patients, doctors, healthcare service providers, and researchers. This study can serve as a guide and help the concerned authorities, such as doctors and researchers, by easing the process to a certain extent when using LLMs and ChatGPT appropriately. When using these LLMs and ChatGPT, their limitations must be considered to ensure accurate results.
